# Towards a barnacle tree of life: integrating diverse phylogenetic efforts into a comprehensive hypothesis of thecostracan evolution

**DOI:** 10.7717/peerj.7387

**Published:** 2019-08-16

**Authors:** Christine Ewers-Saucedo, Christopher L. Owen, Marcos Pérez-Losada, Jens T. Høeg, Henrik Glenner, Benny K.K. Chan, Keith A. Crandall

**Affiliations:** 1 Zoological Museum, Christian-Albrechts University, Kiel, Germany; 2 Systematic Entomology Laboratory, USDA-ARS, Beltsville, MD, USA; 3 Computational Biology Institute, Milken Institute School of Public Health, George Washington University, Ashburn, VA, USA; 4 Department of Invertebrate Zoology, US National Museum of Natural History, Smithsonian Institution, Washington, DC, USA; 5 CIBIO-InBIO, Centro de Investigação em Biodiversidade e Recursos Genéticos, Universidade do Porto, Vairão, Portugal; 6 Marine Biology Section, Department of Biology, University of Copenhagen, Copenhagen, Denmark; 7 Marine Biodiversity Group, Department of Biology, University of Bergen, Bergen, Norway; 8 Biodiversity Research Center, Academia Sinica, Taipei, Taiwan

**Keywords:** Barnacles, Thecostraca, Synthesis tree, Open tree of life, Phylogenetic studies, Taxonomy, Morphology

## Abstract

Barnacles and their allies (Thecostraca) are a biologically diverse, monophyletic crustacean group, which includes both intensely studied taxa, such as the acorn and stalked barnacles, as well as cryptic taxa, for example, Facetotecta. Recent efforts have clarified phylogenetic relationships in many different parts of the barnacle tree, but the outcomes of these phylogenetic studies have not yet been combined into a single hypothesis for all barnacles. In the present study, we applied a new “synthesis” tree approach to estimate the first working Barnacle Tree of Life. Using this approach, we integrated phylogenetic hypotheses from 27 studies, which did not necessarily include the same taxa or used the same characters, with hierarchical taxonomic information for all recognized species. This first synthesis tree contains 2,070 barnacle species and subspecies, including 239 barnacle species with phylogenetic information and 198 undescribed or unidentified species. The tree had 442 bifurcating nodes, indicating that 79.3% of all nodes are still unresolved. We found that the acorn and stalked barnacles, the Thoracica, and the parasitic Rhizocephala have the largest amount of published phylogenetic information. About half of the thecostracan families for which phylogenetic information was available were polyphyletic. We queried publicly available geographic occurrence databases for the group, gaining a sense of geographic gaps and hotspots in our phylogenetic knowledge. Phylogenetic information is especially lacking for deep sea and Arctic taxa, but even coastal species are not fully incorporated into phylogenetic studies.

## Introduction

**Figure 1:**
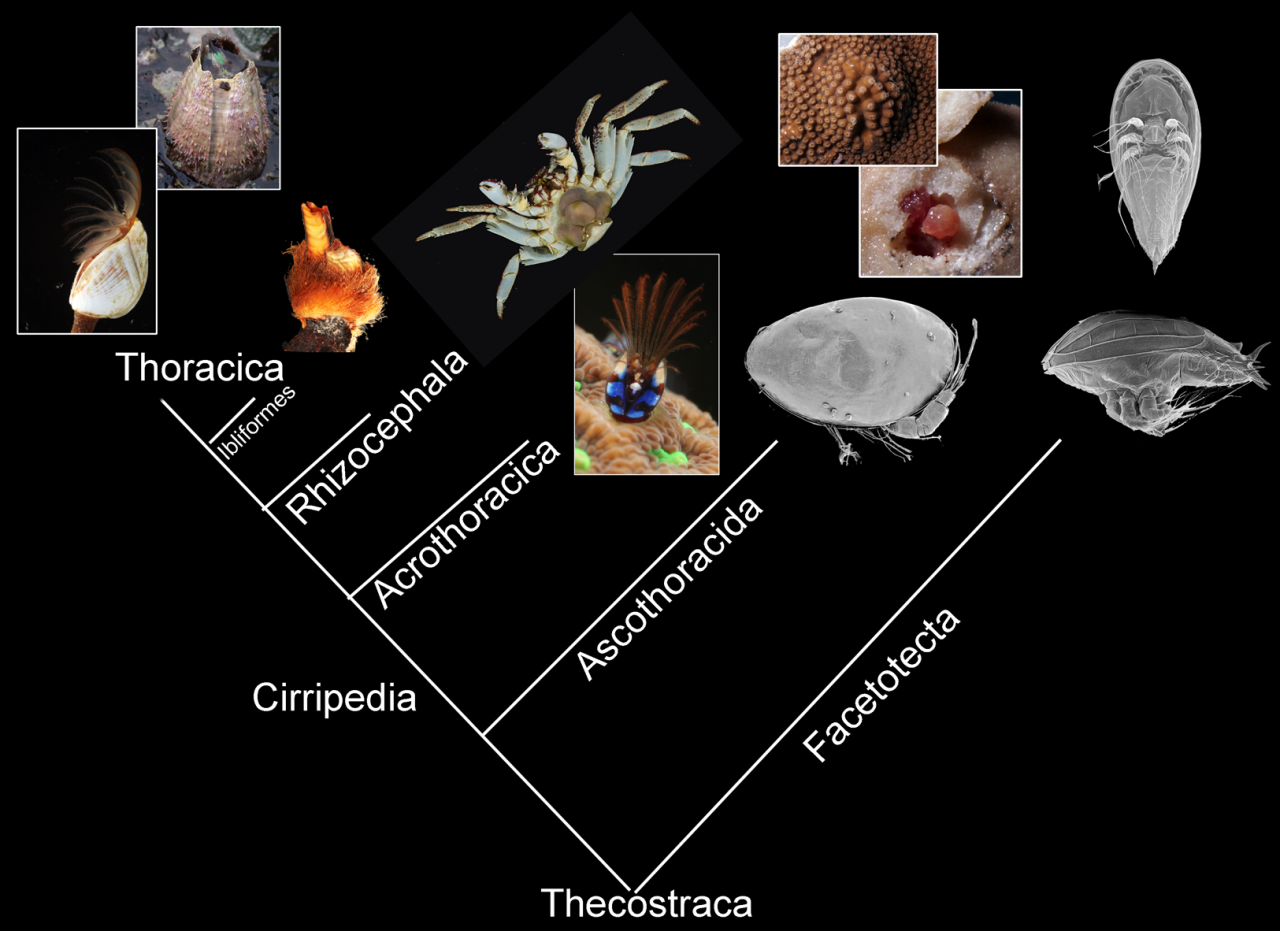
Morphological diversity of Thecostraca mapped onto the phylogenetic hypothesis presented by Pérez-Losada, Høeg & Crandall (2009). The Ibliformes are highlighted as the most basal Thoracican order with several potentially plesiomorphic features. Photographs taken by Benny K.K. Chan.

The Thecostraca, which include not only the barnacles (Cirripedia), but also the Facetotecta, Ascothoracida, and possibly the Tantulocarida (Petrunina et al., 2014), is a highly variable crustacean group in terms of both morphology and biology (Ruppert, Fox & Barnes, 2003; Høeg & Møller, 2006) (Fig. 1). This makes them prime models for studying evolutionary adaptations in diverse fields including morphology, ontogeny, and reproductive systems (Charnov, 1987; Høeg et al., 2009; Yusa et al., 2012; Lin et al., 2015). In fact, the specializations in adult morphology, growth, feeding biology, and sexual systems prompted Darwin (1851a, 1851b, 1854, 1855) to study barnacles, resulting in one of the first “model organisms” of evolutionary adaptation. Recent work has assessed phylogenetic relationships among the barnacles at both the higher (Buckeridge, 1996; Korn, 1995; Harris et al., 2000; Pérez-Losada et al., 2002, 2008; Pérez-Losada, Høeg & Crandall, 2004; Glenner & Hebsgaard, 2006; Mallatt & Giribet, 2006; Høeg et al., 2009) and lower (Mokady et al., 1999; Simon-Blecher, Huchon & Achituv, 2007; Shemesh et al., 2009; Brickner, Simon-Blecher & Achituv, 2010; Pérez-Losada et al., 2014; Lin et al., 2015; Gale, 2018; Lin, Kobasov & Chan, 2016) taxonomic ranks. These studies have provided great insight into barnacle evolution, confirming morphological patterns in some cases, and highlighting substantial convergence in others. For example, genetic data revealed a case of deep convergence in the metamorphosing stages of Rhizocephala and Facetotecta (Pérez-Losada, Høeg & Crandall, 2009), with morphologically very similar slug-shaped stages. In another case, the basal position of Ibliformes within the acorn and stalked barnacles (Thoracica) was confirmed both by the presence of several plesiomorph shell characters and molecular phylogenetics (Høeg et al., 2009; Pérez-Losada et al., 2008). Within the monophyletic Thoracica, the Sessilia (acorn barnacles) are nested within the Pedunculata (stalked barnacles), and the stalk has been lost more than once (e.g., *Neoverrucca*), rendering Pedunculata polyphyletic (Pérez-Losada et al., 2008). Genetic studies confirmed the morphologically suggested sister relationship between Balanomorpha and Verruccomorpha (Pérez-Losada et al., 2008). The often-hypothesized gradual increase of shell plate numbers during the evolution of Thoracica, on the other hand, could not be confirmed (Pérez-Losada et al., 2008). As a consequence of the diverse phylogenetic efforts, few studies have included enough of the same taxa or used the same characters to allow an estimate of the Barnacle Tree of Life up to now.

The inability to combine studies into a single phylogenetic tree for all barnacles is primarily because studies did not include the same species, which is required by supertree methodology, or did not use the same characters (genes or morphology), which is required by supermatrix approaches. Supertree approaches code phylogenies and their represented relationships in a new matrix to be analyzed by phylogenetic methods, and they typically require that a significant number of the same taxa are present in each study to effectively integrate multiple phylogenies into a single tree (for a review see Bininda-Emonds, 2004). Supermatrix approaches, on the other hand, require the same character sets (e.g., nucleotides, proteins, morphological characters, etc.) to be used by each study, and usually contain large amounts of missing data (Driskell et al., 2004; Ciccarelli et al., 2006; McMahon & Sanderson, 2006). This is especially problematic with morphological characters as very different characters have been used at the higher taxonomic ranks compared to lower ranks, and in some groups (e.g., parasitic barnacles) it is impossible to determine character homology. Within Thecostraca, larval characters are the only ones that can be compared across all taxa, but compiling and coding such information is cumbersome and time consuming, especially for rare and hard to sample species (e.g., Yorisue et al., 2016). Similarly, genetic data sets are also hard to combine since experts tend to use different (sometimes completely different) sets of genetic markers. Given the relative lack of matching data across barnacle studies, we have decided to apply a new “synthesis” tree approach (Hinchliff et al., 2015) in an attempt to estimate the first working Barnacle Tree of Life. The synthesis tree approach (Hinchliff et al., 2015; Redelings & Holder, 2017) maps phylogenetic hypotheses onto underlying hierarchical taxonomic information (Rees & Cranston, 2017). This results in an integration of both phylogenetic and taxonomic information onto a single phylogenetic tree that combines phylogenetic relatedness and taxonomic knowledge. This approach also readily highlights those areas of the taxonomy that lack previously published phylogenetic information. Because the phylogenetic information is incorporated as is (i.e., there is no “supermatrix” construction and no re-estimation), any phylogenetic hypothesis can be incorporated regardless of its data basis, hence morphological and/or molecular trees can be integrated without a need for congruent character sets or gene regions.

Given the recent and diverse phylogenetic and genomic efforts across the barnacles, we felt that now was a particularly opportune time to summarize the barnacle phylogeny efforts using phylogenetic synthesis. Indeed, reasonably detailed molecular based trees are now available for most of the major thecostracan groups (Rhizocephala: Glenner & Hebsgaard, 2006; Glenner et al., 2010; Thoracica: Pérez-Losada et al., 2008, 2014; Lin et al., 2015; Chan et al., 2017; Acrothoracica: Lin, Kobasov & Chan, 2016), and several studies have dealt with lower taxonomic ranks (e.g., coral barnacles: Mokady et al., 1999; Simon-Blecher, Huchon & Achituv, 2007; Brickner, Simon-Blecher & Achituv, 2010; Chen, 2012; Tsang et al., 2014; Simon-Blecher et al., 2016). Our goal is to summarize all previously published barnacle phylogenies to highlight areas for future systematic research effort by quickly identifying areas of the taxonomy that lack phylogenetic information as well as areas where there are (1) conflicting phylogenetic hypotheses and/or (2) conflicting phylogenetic and taxonomic information (i.e., non-monophyletic taxa). Additionally, a synthesis tree can also couple taxonomic/systematic information with geographic information and taxa distributions and thereby quickly pinpoint geographic areas for future collecting efforts to add genetic or morphological data to the leaves of the Barnacle Tree of Life that are only represented by taxonomy. Thus, our study demonstrates both the utility of the phylogenetic synthesis approach for obtaining a comprehensive understanding of the state of phylogenetic knowledge for a particular group, and the utility of taxonomy to add geographic information to dark parts of the tree to identify areas for future collecting efforts to complement existing phylogenetic information. Ultimately, this phylogeny serves as the first step to building a comprehensive Barnacle Tree of Life, so hypotheses regarding molecular and morphological barnacle evolution can be further tested.

## Materials and Methods

### Synthesis approach

The two key components in a synthesis phylogeny are first a comprehensive taxonomy of the group in question and second a set of phylogenetic estimates to be integrated with that taxonomy. First, we curated published barnacle phylogenies in the Open Tree of Life online curator (https://tree.opentreeoflife.org/curator) and mapped phylogeny terminals to the underlying taxonomy. We used the open tree taxonomy (OTT) ott2.9 (Rees & Cranston, 2017). The OTT is mainly based on the NCBI Taxonomy from the US National Center on Biotechnology Information (http://www.ncbi.nlm.nih.gov) reference taxonomy, but this taxonomy only includes taxa for which there are molecular data in GenBank. Therefore, to get as complete a taxonomic representation as possible, the NCBI taxonomy has been supplemented with the Backbone Taxonomy from the Global Biodiversity Information facility (www.gbif.org), the World Registry of Marine Species (WoRMS) (http://www.marinespecies.org/) taxonomy, and the Interim Register of Marine and Nonmarine Genera from CSIRO (http://www.cmar.csiro.au/). These taxonomies follow, for the most part, Martin & Davis (2001) for the higher-level classification of the Thecostraca. The backbone taxonomy includes old as well as misspelled species names. These names inflate the number of species (i.e., binomials). We identified these invalid species by matching the tips of the synthesis phylogeny against the well-curated taxonomy of WoRMS and then removed invalid species from the synthesis phylogeny. We did not remove species that could only be identified to the genus level, as these potentially represent valid undescribed species.

If published phylogenies were not available in the Open Tree of Life git-based *phylesystem* repository (McTavish et al., 2015), we surveyed other public repositories and literature for phylogenetic studies on barnacles. For all studies of interest, we searched Supplemental Material, TreeBase (www.treebase.org), DataDryad (www.datadryad.org) and FigShare (https://www.figshare.com/) for files of phylogenetic trees in a re-usable text format (e.g., nexus, newick, phylip, etc.). Unfortunately, the systematics community on average does not treat phylogenetic estimates as digital information to be deposited in an electronic repository (Drew et al., 2013). Therefore, if re-usable tree files were not available, we contacted the authors. If the authors were not able to provide tree files, we manually reconstructed newick trees in Mesquite v.3.40 (Maddison & Maddison, 2018) based on the phylogenetic tree presented in the respective study. These trees do not have meaningful branch lengths. This is acceptable as input for synthesis tree reconstruction because branch length information is not used in the tree synthesis process.

One requirement of phylogenetic synthesis is to rank input phylogenies with the phylogeny that will carry the most weight first and the phylogeny that will carry the least weight last. This means the phylogeny ranked first will have its bifurcations favored over all others ranked below it in the final synthesis phylogeny. Due to this weighting scheme, we ranked the barnacle taxonomy last because we wanted all molecular or morphological input trees ranked ahead of the taxonomy and not taxa or branches represented by molecular or morphological data were represented by taxonomy. For the synthesis tree construction, we ranked molecular and morphological studies by three criteria:

1. Scope: studies with a narrow phylogenetic scope, for example, focused on one or a few genera, were ranked higher than studies with a broad scope. The rationale is that studies focusing on lower taxonomic ranks have a better resolution at the shallow nodes, while broader studies that contain a diversity of higher taxonomic ranks typically add little phylogenetic information at shallow nodes. Giving studies with narrow scope a higher rank means that in case of a conflict, those studies take precedence over the lower-ranked trees.
2. Number of markers: if studies aimed to reconstruct the same most recent common ancestor (mrca), for example, five studies attempting to resolve relationships within the Thoracica (Table 1), we ranked studies with more molecular markers higher. Here, we assume that including more markers leads to better phylogenetic reconstructions, depicting evolutionary relationships more realistically. They are less likely to reconstruct gene trees, and more likely to reconstruct the “true” species tree.
3. Number of taxa: if rankings could not be resolved based on the previous two criteria, we ranked studies including more genera or more species higher. This practice follows the idea that missing taxa can hamper the reconstruction of accurate species relationships (Zwickl & Hillis, 2002).

**Table 1:** Information on the phylogenetic studies used to synthesize the Barnacle Tree of Life.

Rank	Study	Focal taxon	Scope	Markers	# species	# genera	Tree inference method
1	Chan, Chen & Lin (2013a)	*Galkinia*	Genus	12S, COI	8	1	ML
2	Van Syoc et al. (2010)	*Pollicipes*	Genus	COI, H3, 16S	4	1	BI
3	Wares et al. (2009)	*Chthamalus*	Genus	16S, NKA, EF1α	18	2	BI, MP, NJ
4	Carrison-Stone et al. (2013)	*Conopea*	Genus	COI, H3	4	1	BI
5	Glenner et al. (2008)	*Heterosaccus*	Genus	16S, 18S	4	1	BI
6	Glenner, Lützen & Takahashi (2003)	*Polysacus*	Genus	18S, COI	7	1	MP
7	Van Syoc & Newman (2010)	Bryozobiinae	Subfamily	morphology	5	5	MP
8	Mokady et al. (1999)	Pyrgomatinae	Subfamily	12S	5	2	ML, NJ, MP
9	Malay & Michonneau (2014)	Pyrgomatidae	Family	COI, 16S, 12S, 18S, H3, morphology	22	15	BI, ML
10	Tsang et al. (2014)	Pyrgomatidae	Family	12S, 16S, EF1α, H3, RPII	26	11	BI, ML
11	Simon-Blecher, Huchon & Achituv (2007)	Pyrgomatidae	Family	12S, 16S, 18S	27	20	BI, ML
12	Tsang et al. (2015)	Tetraclitidae	Family	12S, 16S, 18S, COI, EF1, H3, RPII	30	8	BI, ML
13	Hayashi et al. (2013)	Coronuloidea	Superfamily	12S, 16S, 18S, 28S, H3	27	19	BI, ML
14	Pérez-Losada et al. (2014)	Balanomorpha	Suborder	12S, 16S, 18S, 28S, COI	124	65	BI, ML
15	Chan et al. (2013b)	Lithoglyptida	Order	16S, COI	5	5	ML, NJ
16	Glenner et al. (2010)	Akentrogonida	Order	18S, 28S	15	11	BI
17	Linse et al. (2013)	Scalpelliformes	Order	18S, 28S, COI	10	6	BI
18	Lin, Kobasov & Chan (2016)	Acrothoracica	Superorder	16S, 18S, COI, H3	22	8	BI
19	Høeg et al. (2019)	Rhizocephala	Superorder	16S, 18S, 28S	27	16	BI, ML
20	Glenner & Hebsgaard (2006)	Rhizocephala	Superorder	18S	22	16	BI, ML
21	Lin et al. (2015)	Thoracica	Superorder	12S, 18S, COI, H3	78	36	BI
22	Rees et al. (2014)	Thoracica	Superorder	16S, 18S, 28S	98	59	BI
23	Herrera, Watanabe & Shank (2015)	Thoracica	Superorder	28S, COI, H3	100	52	ML
24	Pérez-Losada et al. (2008)	Thoracica	Superorder	18S, 28S, H3	76	43	BI, ML
25	Yusa et al. (2012)	Thoracica	Superorder	18S	48	27	BI, ML
26	Pérez-Losada, Høeg & Crandall (2009)	Thecostraca	Subclass	18S, 28S, H3, morphology	79	66	BI, ML
27	Petrunina et al. (2014)	Thecostraca	Subclass	18S	8	8	BI

**Note:**

Rank refers to the order in which a tree of the respective phylogenetic study was included into the synthesis approach. Focal taxon denotes the focal taxonomic unit of the publication. Scope refers to the taxonomic rank of the focal taxon. Markers are the markers used to reconstruct the phylogeny. Morphology refers to any number of morphological characters. All other markers refer to molecular DNA sequences, which amplified a gene or RNA fragment of the mitochondrial or nuclear genome. The mitochondrial markers were either 16S rRNA, and cytochrome oxidase subunit 1 (COI). The nuclear markers were 12S rRNA, 18S rRNA, 28S rRNA, histone 3 gene (H3), Na-K-ATPase (NKA), eukaryotic elongation factor 1α (EF1α), and RNA polymerase subunit II (RPII). In most cases, only a fragment of the RNA or gene was amplified. Tree inference methods: BI, Bayesian inference; ML, maximum likelihood; MP, maximum parsimony; NJ, neighbor-joining.

In addition to requiring ranked barnacle studies, additional software and information is needed to generate a synthesis tree. The synthesis tree was generated using *propinquity* (https://github.com/OpenTreeOfLife/propinquity) (Redelings & Holder, 2017), which requires the following software: *otcetera* v0.0.01 (https://github.com/OpenTreeOfLife/otcetera) and *peyotl* v0.1.4 (https://github.com/OpenTreeOfLife/otcetera). Taxa not represented in the published phylogenies are represented by taxonomy only in the synthesis tree, allowing for identification of conflict between these sources of information and identifying taxa for which phylogenetic information is totally lacking. In the case of conflict between phylogenies and backbone taxonomy, the synthesis tree reflects the phylogeny rankings, not the taxonomy. We used OTT v3.0 (/home/bredelings/Devel/OpenTree/ott-3.0) taxonomy, while setting the root taxon to ott580064. The root taxon corresponds to mrca of the barnacles according to the taxonomy. Lastly, the ranked order of the barnacle studies (Supplemental File), paths to software and taxonomy, and the root taxon were entered into the *propinquity* configuration file and executed using the commands *make* and *make check*.

### Geographic information

For all thecostracan species, we searched for occurrence data using currently accepted taxonomic names as well as their synonyms. For synonyms, we followed WoRMS (Walter & Boxshall, 2016) and Integrated Taxonomic Information System (www.itis.gov/). We extracted occurrence data from the Global Biodiversity Information Facility (GBIF: http://www.gbif.org/, consulted May 2016).

## Results

### Synthesis phylogeny estimation

We identified a total of 36 phylogenetic studies on barnacle evolution. We excluded nine studies for which a subsequent study investigated the same species with more markers and/or additional species (often subsequent studies from the same authors) for a final set of 27 studies (Table 1). For 16 studies, we received the tree files from the authors. Herrera, Watanabe & Shank (2015) had deposited their tree file in DataDryad, Lin et al. (2015) in TreeBase, and Hayashi et al. (2013) provided their tree file as Supplemental Information. For the remaining eight studies, we reconstructed the tree manually in Mesquite based on published figures. All trees are available in the “barnacles tree collection” of the Open Tree of Life online curator (https://tree.opentreeoflife.org/curator/collections/kcranston/barnacles).

**Figure 2:**
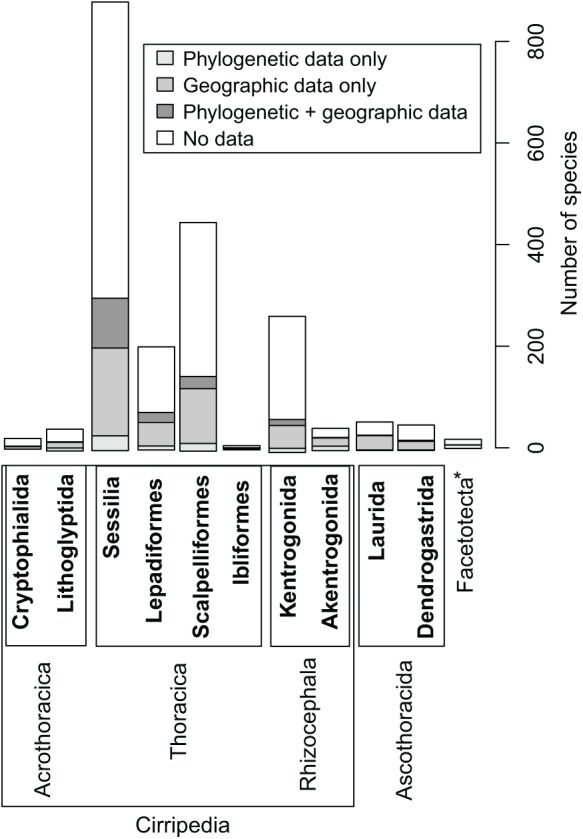
Distribution of phylogenetic and geographic information available across the main thecostracan orders. Bars represent the number of species, with different shades of gray denoting the number of species for which phylogenetic, geographic or both information are available. The asterisk indicates that no orders are defined for the infraclass Facetotecta.

The initial synthesis tree of barnacles (Thecostraca) contained a total of 2,272 tip labels (terminals), of which 202 tip labels were invalid species names (e.g., synonyms or misspellings) that were removed from the synthesis tree. Of the remaining species, 1,872 were described species or subspecies, and 198 were undescribed or unidentified species for a total of 2,070 tree tips. This synthesis tree is available in the supplement of this publication. Phylogenetic information was available for 239 described species (11.5% of all barnacle species). This information was not evenly distributed among barnacle orders (Fig. 2). The order Sessilia had the highest absolute coverage, with 127 of 883 species (14.4%) being represented in phylogenetic studies. The small order Ibliformes had the highest relative coverage (25%) with two out of eight species (Fig. 2). The other two orders of Thoracica, the Lepadiformes (203 species) and Scalpelliformes (450 species), were represented in phylogenetic studies by 27 and 34 species, respectively. The two orders of Rhizocephala (311 species), the Akentrogonida (43 species) and Kentrogonida (268 species), were relatively well represented in phylogenetic studies with 10 and 19 species, respectively. The Acrothoracica (71 species) are overall species-poor; its orders Cryptophialida (21 species) and Lithoglyptida (50 species) were represented in phylogenetic studies with two and 11 species, respectively. The two orders of Ascothoracida (106 species), the Dendrogastrida (50 species) and Laurida (56 species), are also relatively small, and only five ascothoracidan species were used in phylogenetic studies to understand the position of Ascothoracida at large. The enigmatic Facetotecta and Tantulocarida were represented by one and two species, respectively.

**Figure 3:**
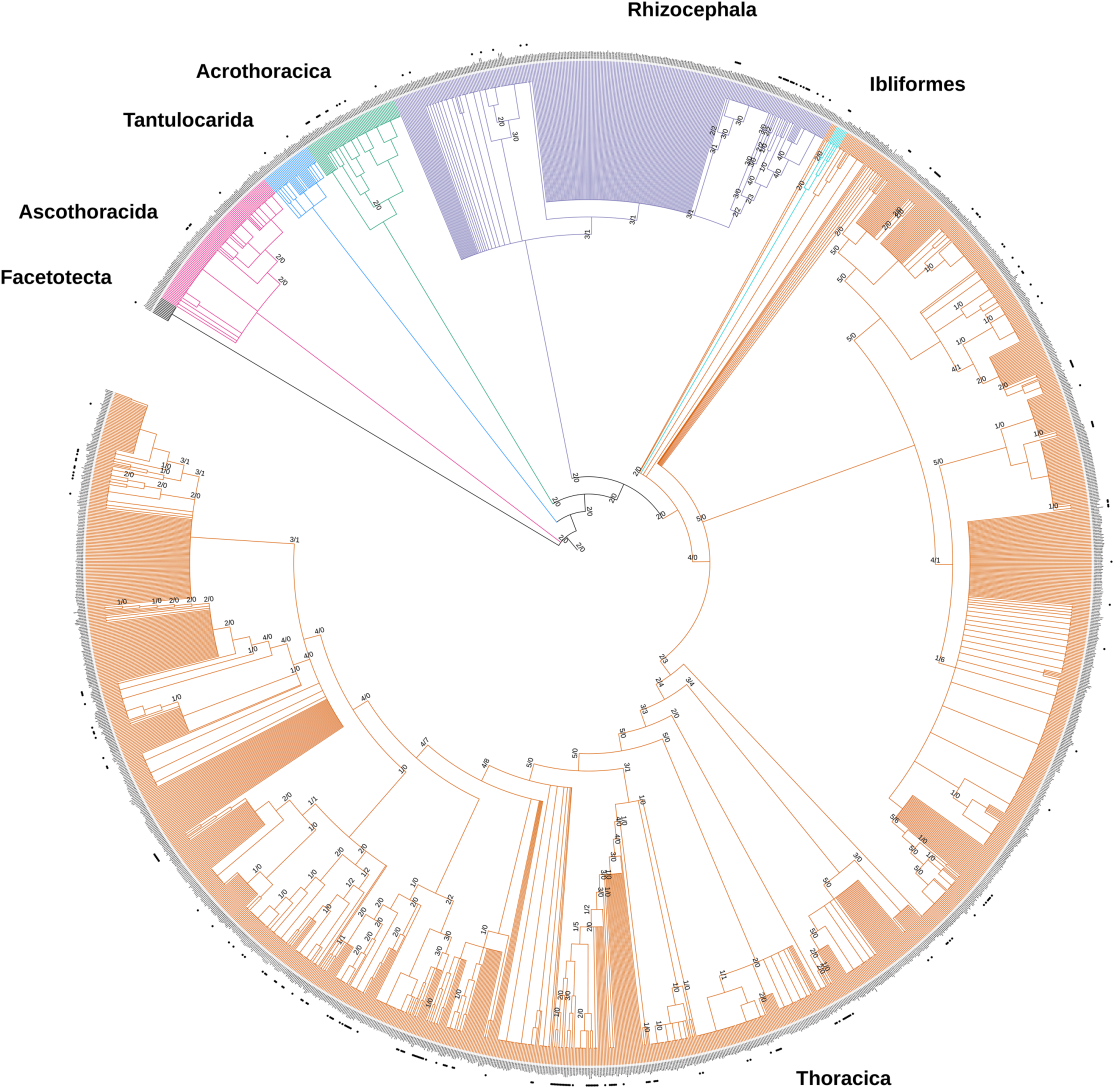
Synthesis phylogeny of all thecostracan species. Species with phylogenetic information have a black dot adjacent to their names. Higher thecostracan taxonomy is colored and labeled accordingly (matching the taxonomic units presented in Fig. 1). Branch support/conflict values are plotted onto the branches. The first number indicates the number of input trees that support the branch, and the second number indicates the number of trees that conflict with the tree synthesis.

A completely resolved (bifurcating) tree would have 2,135 nodes, but the synthesis tree has only 442 bifurcating nodes, indicating that 79.3% of all nodes are unresolved. This is also apparent in the visualization of the synthesis tree (Fig. 3), where most nodes are polytomous. The tree visualization with its annotations (including the later described node support values) can be reconstructed via the interactive Tree of Life website (https://itol.embl.de/) (Letunic & Bork, 2019). We provide the synthetic tree and text files containing the tree annotations as Supplemental Material. After creating an iTOL account and uploading the tree to the website, the annotation files can be dragged and dropped onto the tree image. Polytomies are caused by missing phylogenetic information and indicates that the node is supported by taxonomic information alone. Our source trees provide phylogenetic information for 220 internal nodes. Of the 220 nodes, 191 have more support than conflict, 18 have more conflict than support, and 11 have equal number of supporting and conflicting source phylogenies (Fig. 3). The most conflicted node is the mrca of a clade containing e.g., *Amphibalanus improvisus* and *Tetraclita japonica formosana*, which contains 41 genera and 419 terminal taxa. *Amphibalanus improvisus* and *Tetraclita japonica formosana* are taken as representatives of this clade, but have not been used in phylogenetic reconstructions. For the node in question, there are eight phylogenies in conflict and four phylogenies that support the relationships in Fig. 3. The largest number of source phylogenies supporting an internal node is five and there are 16 nodes in the synthesis tree with five source trees supporting a node.

Phylogenetic information on more than one species was lacking for 19 out of 56 families, so we were unable to assess the monophyly of those taxa (Table 2). Of 38 families with phylogenetic information, 18 were monophyletic. All orders but the small Ibliformes were polyphyletic. Polyphylies are also prevalent at the lower taxonomic ranks, such as the genus level. These polyphyletic genera caused a large number of thecostracan barnacle species to be placed basally with regard to their congeners. Those species were not included into phylogenetic studies, but some of their congeners were. Those congeners revealed that the genus or higher taxonomy in question were not monophyletic, thus making it impossible to place the remaining congeners solely based on taxonomy. The genera *Trianguloscalpellum* and *Arcoscalpellum*, for example, are polyphyletic, leading to an accumulation of species of those two genera at the base of the Scalpellidae (Fig. 3). This broken taxonomy can only be fixed by taxonomic revisions that are congruent with current phylogenetic results. Only monophyletic taxa allow the placement of all members of a genus (or higher taxonomy) into the same branch, as is the case for the genus *Scalpellum*.

**Table 2:** Number of species in each family and the number and proportion (in parentheses) of species for which phylogenetic data or geographic information is available.

Family	Higher taxa	Total number of species	Phylogenetic data	Geographic information
Anelasmatidae	Thoracica	1	1 (1.00)	1 (1.00)
Archaeobalanidae	Thoracica	161	8 (0.05)	29 (0.18)
Ascothoracidae	Ascothoracida	9	0 (0.00)	3 (0.33)
Austrobalanidae	Thoracica	18	4 (0.28)	8 (0.44)
Balanidae	Thoracica	202	14 (0.10)	82 (0.41)
Calanticidae	Thoracica	61	7 (0.15)	22 (0.36)
Catophragmidae	Thoracica	3	2 (0.67)	1 (0.33)
Chelonibiidae	Thoracica	9	5 (0.44)	5 (0.56)
Chionelasmatidae	Thoracica	3	0 (0.00)	1 (0.33)
Chthamalidae	Thoracica	67	33 (0.48)	24 (0.36)
Chthamalophilidae	Rhizocephala	4	2 (0.75)	3 (0.75)
Clistosaccidae	Rhizocephala	2	1 (0.50)	2 (1.0)
Coronulidae	Thoracica	13	2 (0.31)	4 (0.31)
Cryptophialidae	Acrothoracica	21	1 (0.10)	6 (0.29)
Ctenosculidae	Ascothoracida	3	0 (0.00)	3 (1.00)
Dendrogastridae	Ascothoracida	38	3 (0.08)	12 (0.32)
Duplorbidae	Rhizocephala	5	0 (0.00)	2 (0.40)
Eolepadidae	Thoracica	53	5 (0.08)	3 (0.06)
Heteralepadidae	Thoracica	57	5 (0.09)	13 (0.23)
Iblidae	Thoracica	3	2 (0.67)	2 (0.67)
Idioiblidae	Thoracica	5	0 (0.00)	2 (0.40)
Koleolepadidae	Thoracica	4	1 (0.25)	1 (0.25)
Lauridae	Ascothoracida	18	1 (0.06)	3 (0.17)
Lepadidae	Thoracica	27	8 (0.30)	11 (0.41)
Lernaeodiscidae	Rhizocephala	17	1 (0.06)	6 (0.35)
Lithoglyptidae	Acrothoracica	33	9 (0.03)	13 (0.39)
Lithotryidae	Thoracica	6	3 (0.50)	2 (0.33)
Malacolepadidae	Thoracica	1	0 (0.00)	0 (0.00)
Microlepadidae	Thoracica	3	0 (0.00)	0 (0.00)
Mycetomorphidae	Rhizocephala	2	1 (0.00)	1 (0.50)
Neoverrucidae	Thoracica	30	3 (0.10)	0 (0.00)
Oxynaspididae	Thoracica	29	2 (0.07)	4 (0.14)
Pachylasmatidae	Thoracica	52	3 (0.06)	8 (0.15)
Parthenopeidae	Rhizocephala	2	1 (0.50)	1 (0.50)
Peltogastridae	Rhizocephala	45	4 (0.09)	12 (0.27)
Petrarcidae	Ascothoracida	11	1 (0.09)	8 (0.73)
Platylepadidae	Thoracica	24	9 (0.38)	8 (0.33)
Poecilasmatidae	Thoracica	70	10 (0.16)	30 (0.43)
Pollicipedidae	Thoracica	7	3 (0.43)	4 (0.57)
Polysaccidae	Rhizocephala	2	1 (0.50)	0 (0.00)
Pyrgomatidae	Thoracica	119	21 (0.13)	17 (0.14)
Rhizolepadidae	Thoracica	2	0 (0.00)	0 (0.00)
Sacculinidae	Rhizocephala	196	13 (0.07)	34 (0.17)
Scalpellidae	Thoracica	310	16 (0.07)	89 (0.29)
Synagogidae	Ascothoracida	27	0 (0.00)	18 (0.67)
Tetraclitidae	Thoracica	50	19 (0.26)	24 (0.48)
Thompsoniidae	Rhizocephala	25	5 (0.12)	1 (0.04)
Trypetesidae	Acrothoracica	7	2 (0.00)	4 (0.57)
Verrucidae	Thoracica	81	7 (0.10)	36 (0.44)

### Geographic analysis

**Figure 4:**
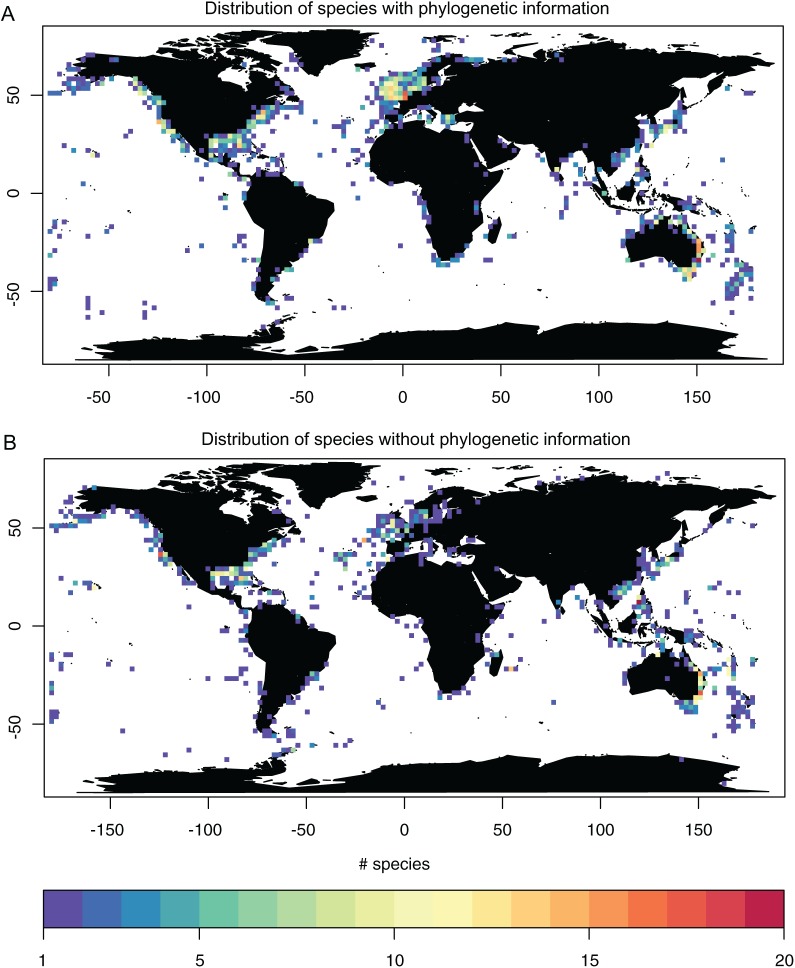
Geographic occurrence of thecostracan species with (A) and without (B) phylogenetic information, based on publicly available geographic occurrence records (Global Biodiversity Information Facility, www.gbif.org).

Geographic information system (GIS) occurrence information was available for 596 species (Supplemental Table). Of those, 111 species were represented in phylogenetic studies, many of which belonged to the most frequently geo-referenced species. Species with few geo-references, on the other hand, were less often represented in phylogenetic studies. Exceptions are represented in Table 3. Comparing the distribution of taxa with and without geographic information reveals that the coasts of the USA, Europe and Australia have the highest density of records, both of species with and without phylogenetic information (Fig. 4). The deep sea and Antarctica, on the other hand, have very few records. Europe has the highest number of geo-referenced species that have also been sampled for phylogenetic studies (Fig. 4A), while species not yet included into phylogenetic studies are found along all coasts (Fig. 4B).

**Table 3:** Species with more than 30 geographic occurrence records (downloaded from www.gbif.org) but without phylogenetic data.

Species	GBIF records
*Amphibalanus improvisus*	1,035
*Arcoscalpellum michelottianum*	131
*Striatobalanus amaryllis*	119
*Balanus rostratus*	86
*Trypetesa spinulosa*	74
*Meroscalpellum bifurcatum*	72
*Octolasmis orthogonia*	69
*Tesseropora rosea*	66
*Pollicipes elegans*	63
*Capitulum mitella*	56
*Neoscalpellum debile*	53
*Austrobalanus imperator*	50
*Arcoscalpellum portoricanum*	47
*Trypetesa lateralis*	42
*Acasta spongites*	38
*Anguloscalpellum pedunculatum*	33
*Acasta cyathus*	32
*Balanus laevis*	32
*Chionelasmus darwini*	32
*Notomegabalanus algicola*	32
*Armatobalanus quadrivittatus*	31
*Peltogaster paguri*	31
*Striatobalanus tenuis*	31

## Discussion

Barnacles were one of the first model systems used in evolutionary biology (Darwin, 1851a, 1851b, 1854, 1855), and have remained important in evolutionary (Charnov, 1987; Høeg & Møller, 2006; Høeg et al., 2009; Kelly & Sanford, 2010; Yusa et al., 2012), developmental (Mouchel-Vielh et al., 1998; Høeg, Chan & Semmler, 2015; C Ewers-Saucedo & P Pappalardo, 2017, unpublished data), and ecological studies (Dayton, 1971; Grosberg, 1982; Shinen & Navarrete, 2010; Lamb, Leslie & Shinen, 2014). All these fields can benefit from phylogenetic information to account for the non-independence of species, and to unveil macroevolutionary patterns (Høeg, 1995; Pérez-Losada, Høeg & Crandall, 2009; Glenner et al., 2010; Rees et al., 2014; Lin et al., 2015; C Ewers-Saucedo & P Pappalardo, 2017, unpublished data). In the present study, we curated the available phylogenetic and taxonomic information for barnacles and reconstructed a complete synthesis phylogeny.

Recent phylogenetic efforts have investigated all major groups in the thecostracan tree. A total of 20 of the 27 studies we included focused on the Thoracica, the acorn and stalked barnacles (Table 1). This is not surprising, as these predominantly free-living barnacles are omnipresent in marine habitats, ecologically important and economically-costly fouling organisms. Rhizocephala are also relatively well-represented in phylogenetic studies. These specialized parasites of crabs and other economically-important crustaceans provide interesting model systems for development and host manipulation (Kobayashi et al., 2018). Fewer studies have considered the placement of the enigmatic Facetotecta, of which only the larvae are known, the Ascothoracida, ectoparasites of cnidarians and echinoderms (Pérez-Losada, Høeg & Crandall, 2009), and the shell-boring Acrothoracica (Lin, Kobasov & Chan, 2016). The number of species in a taxon and its phylogenetic coverage appear to be linked—that is, less well-studied taxa contain fewer species. We hypothesize that these taxa contain much cryptic diversity, which has remained hidden to date. This hypothesis is supported by the findings of the first comprehensive molecular phylogeny of Acrothoracica (Lin, Kobasov & Chan, 2016), which included 11 described species, and identified an additional 12 cryptic operational taxonomic units. This suggests that species diversity in the Acrothoracica could be twice as high as current species numbers indicate.

An unexpected result of our study is that even in geographic regions with a long history of marine research, such as the coasts of Europe, the United States and Australia, not all barnacle species have been included in phylogenetic analyses, and some of the most common species are lacking phylogenetic information, e.g., *Amphibalanus improvisus* (see Table 3 for more examples). Less surprising is the fact that remote regions, such as the open ocean and the Arctic coast, are under-sampled for many taxa, both with regard to phylogenetic and geographic information. While it should be relatively easy to include all barnacle species from marine biology hotspots into future phylogenetic studies, the under-sampling issue requires larger effort. However, remote regions potentially contain much of the existing phylogenetic diversity (Newman & Ross, 1971) and could provide novel insights into the evolution of barnacles.

Our comparison of phylogenetic and taxonomic hypotheses revealed that many families were polyphyletic. These polyphylies lead to the accumulation of species from polyphyletic taxa at the base of the barnacle tree: all species that belong to polyphyletic taxa but have not themselves been included into phylogenetic studies can only be placed at the next higher taxonomic rank. The most extreme case of this “broken taxonomy” are the genera *Pseudoacasta*, *Zulloana*, *Hexacreusia*, *Eoatria*, *Multatria*, *Microporatria*, *Bryozobia*, *Poratria*, and *Membranobalanus*. These genera are placed at the very base of the Thoracica, next to the “real” sister taxon to the remaining Thoracica, the Ibliformes. They are, by no means, basal genera of the Thoracica, and their placement is an artefact of the tree synthesis. All of these genera belong to the Archaeobalanidae, a taxon that is highly polyphyletic. This disparity between phylogeny and taxonomy is likely caused by the use of morphological character sets to define taxonomy vs. molecular characters used to estimate phylogeny. Furthermore, many barnacle taxa are still defined based on symplesiomorphic similarity or their classification relies on characters highly prone to homoplasy (Pérez-Losada et al., 2014; Gale, 2018). While there has been a robust debate on the relative merits of molecular vs. morphological characters for estimating phylogenies, molecular characters have been especially useful to solve barnacle systematics. Within a morphologically-diverse taxon such as the barnacles, morphological characters may not be homologous, which further complicates the use of morphological data (Gale, 2016). Additionally, larval characters are the only morphological datasets that can be compared across all thecostracan taxa, but compiling them is difficult and time consuming (Høeg et al., 2009). To address the discrepancies between taxonomy and phylogenies, a thorough revision of the barnacle taxonomy is in order. To improve taxonomic assessments in the absence of molecular data, morphological synapomorphies that are congruent with molecular phylogenetic reconstructions should be identified (e.g., Høeg et al., 2009; Lin et al., 2015; Gale, 2018 and references therein). These characters may then be applied to taxa for which molecular data cannot be obtained at present, especially rare species, and species from remote areas of the world, such as the deep sea and arctic regions. Extending the molecular-based trees using morphology is also crucial for integrating fossil information, which in barnacles offers an extensive and well-preserved set of taxa and characters (Pérez-Losada et al., 2008; Gale, 2018). It is also promising to see that larval characters studied at the ultrastructural level almost always match closely with molecular phylogenies (Høeg & Kolbasov, 2002; Glenner et al., 2010).

Although we now have a working rendition of the Barnacle Tree of Life, much work is needed to confirm relationships among higher taxa and lower ranks. For example, the Superorder relationships have been supported by molecular and morphological data, but the barnacle phylogeny would benefit from a phylogeny of higher taxa based on genomic data. While currently there are 58 thecostracan transcriptomes on NCBI SRA database (last accessed March 13, 2018), no phylogenomic phylogenetic estimate has been generated yet. The taxonomic coverage of the available transcriptomes primarily covers Orders within Thoracica (Sessilia and Pedunculata), while one transcriptome is available for the Superorder Rhizocephala (Order Kentrogonida). A phylogenomic estimate for the Thecostraca would require obtaining additional samples for representatives in the Superorder Acrothoracica, Ascothoracida, Facetotecta, and Tantulocarida (whose taxonomic position is still questionable). It should further be noted that no barnacle genomes are available despite their relatively small genome sizes of 0.67–2.60 *C*-value (Gregory, 2018).

Lastly, we would like to highlight the benefits of making phylogenetic trees available for further systematic research. The OTL project provides a user-friendly interface to upload trees and metadata to the OTL workflow (https://tree.opentreeoflife.org/curator). As we have done here, uploaded trees can be combined into a larger phylogenetic framework. This can help answer taxonomic questions, guide future phylogenetic efforts and allow the inclusion of a large number of species into comparative studies. To date, comparative studies have often been limited by the availability of phylogenetic information. Lin et al. (2015), for example, reconstructed a phylogeny for 77 barnacle species with various sexual systems, and mapped the evolution of sexes onto this tree. C Ewers-Saucedo & P Pappalardo (unpublished data), on the other hand, utilized the Barnacle Tree of Life to map all available larval trait data onto the thoracican tree, which allowed the inclusion of 170 thoracican species and did not require the collection of additional phylogenetic information.

## Conclusions

This study provides the first working Barnacle Tree of Life, based on the phylogenetic information of 27 studies and a comprehensive taxonomic backbone. This tree highlights large gaps in our knowledge of barnacle phylogenetics, both with regard to taxonomy as well as geographic sampling. Nonetheless, this tree is a first working hypothesis for all barnacle species and provides therefore a valuable resource for comparative studies. The iterative nature of the OTL project allows—and is fueled by—the inclusion of future phylogenetic studies, which will continuously expand and improve the Barnacle Tree of Life.

## Supplemental Information

10.7717/peerj.7387/supp-1 Supplemental Information 1 Geographic occurrence records of thecostracan barnacles available in the Global Biodiversity Information Facility (GBIF) database (https://www.gbif.org/). All records for thecostracan barnacles were downloaded in May 2016. Each row denotes one record. One species can have many records.

10.7717/peerj.7387/supp-2 Supplemental Information 2 Synthesis phylogeny of thecostracan barnacles. This tree synthesizes phylogenetic information from 27 studies (see Table 1 for details) as well as a backbone taxonomy where phylogenetic information is lacking.

10.7717/peerj.7387/supp-3 Supplemental Information 3 Original output files of the tree synthesis. This zip folder contains the initial tree, the annotations, files with branch support and a tree built only with the species for which phylogenetic information is available.

10.7717/peerj.7387/supp-4 Supplemental Information 4 Input order of phylogenetic studies for synthesis pipeline. Ranked study identifiers used as input for the *propinquity* pipeline

10.7717/peerj.7387/supp-5 Supplemental Information 5 ITOL tree annotations. Several text files that can be dragged and dropped onto the image of the synthetic tree in iTOL. The files contain labels and colors used to make Fig. 3, as well as support values and labels for all monophyletic families.

## Additional Information and Declarations

### Data Availability

The following information was supplied regarding data availability: We generated, curated and analysed a collection of phylogenetic trees available at the Open Tree of Life Curator tool: https://tree.opentreeoflife.org/curator/collections/kcranston/barnacles.
